# Prognostication of prostate cancer based on TOP2A protein and gene assessment: TOP2A in prostate cancer

**DOI:** 10.1186/1479-5876-11-36

**Published:** 2013-02-11

**Authors:** Marina França de Resende, Samantha Vieira, Ludmilla Thomé Domingos Chinen, Francesco Chiappelli, Francisco Paulo da Fonseca, Gustavo Cardoso Guimarães, Fernando Augusto Soares, Ivan Neves, Simone Pagotty, Peter A Pellionisz, Andre Barkhordarian, Xenia Brant, Rafael Malagoli Rocha

**Affiliations:** 1 Department of Pathology, A. C. Camargo Cancer Hospital, Sao Paulo, Brazil; 2 UCLA School of Dentistry, Los Angeles, CA, USA; 3 Department of Urology, A. C. Camargo Cancer Hospital, Sao Paulo, Brazil; 4 Rua Professor Antônio Prudente 211, Liberdade São Paulo, SP, 01509-900, Brazil

**Keywords:** FISH, Immunohistochemistry, Prostate cancer, TOP2A

## Abstract

**Background:**

*TOP2A* encodes for topoisomerase IIα, a nuclear enzyme that controls DNA topological structure and cell cycle progression. This enzyme is a marker of cell proliferation in normal and neoplastic tissues; however, little information is available about its expression in prostate cancer (PCa).

**Methods:**

Immunohistochemistry (IHC) was automated using mouse monoclonal antibody against TOP2A (clone SWT3D1; DAKO, Carpenteria, CA, USA) at dilution 1:800 and Flex Plus detection system in autostainer 48Ultra (DAKO). FISH was performed using TOP2A (17q21)/ CEP17 probe kit (Kreateck Biotechnology, San Diego, CA, USA). Biochemical and pathological data from 193 patients with PCa were retrieved for the analysis, whose significance was considered when p < 0.05. Also, fractal analysis was performed in a subset of 20 randomly selected cases.

**Results:**

TOP2A protein expression correlated with higher Gleason scores and higher levels of preoperative PSA (*p* = 0.018 and *p* = 0.011). Patients with higher levels of TOP2A presented shorter biochemical recurrence-free survival (BRFS) (*p* = 0.001). In multivariate analysis, we found that TOP2A remained an independent prognostic factor of BRFS, with a relative risk of 1.98 (*p* = 0.001; 95% CI, 1.338–2.93); thus, cases that expressed high levels of this enzyme had a shorter BRFS compared with TOP2A-negative or TOP2A-low cases. No alterations in *TOP2A* gene status nor correlation between FISH and IHC results were observed. Concerning fractal analysis, patients who expressed higher levels of TOP2A have angiolymphatic invasion and presented higher Gleason scores (*p* = 0.033 and *p* = 0.025, respectively). Also, patients with higher expression of TOP2A presented shorter BRFS (*p* = 0.001).

**Conclusions:**

This is the first study to perform TOP2A protein and gene digital assessment and fractal analysis in association with BRFS in a large series of PCa. Also, we show that *TOP2A* gene copy number alterations are not observed in this type of tumor. So, higher protein expression of TOP2A is not related to gene amplification in PCa. Furthermore, TOP2A protein assessment has prognostic importance and, due to its relation with poor outcome, TOP2A IHC evaluation in the biopsy can represent an important tool for selecting the most suitable surgical and clinical approach for patients with PCa.

## Introduction

Two homologous but distinct isoforms of type II human topoisomerases have been identified: the 1531 amino acid 170 kDa DNA topoisomerase IIα (TOP2A) and the 1621 amino acid 180 kDa DNA topoisomerase IIβ (TOP2B) encoded by the highly related *TOP2A* and *TOP2B* genes, respectively [1,2]. TOP2A plays important roles in DNA synthesis and transcription, as well as chromosomal segregation during mitosis [1]. Beyond its physiological functions, TOP2A is reported to be a sensitive and specific marker of actively proliferating cells (in the late S, G_2_ and M-phases of the cell cycle), which suggests the importance of its investigation in cancer [1].

Among men, cancer of the prostate, lung and bronchus, and colorectum accounted for 52% of all newly diagnosed cancers in 2010 [3]. Generally, prostate cancer (PCa) alone accounts for 28% (217,730) of incident cases in men [3]. This disease exhibits considerable variability in clinical behavior [4]. Many (if not most) PCa are clinically indolent, while others are clinically aggressive, becoming metastatic and lethal [4]. For localized PCa, treatment options range from active surveillance to decisive surgical excision (radical prostatectomy) or radiation therapy [4]. Increasingly, there is a need for prognostic biomarkers to accurately stratify patients for appropriate risk-adapted therapy [4]. TOP2A is clearly a proliferation marker and proliferation measurements in PCa have repeatedly been shown to provide prognostic information [4-11]. However, little information is available about TOP2A expression in prostate carcinoma [6].

In the present study we showed the prognostic importance of TOP2A in PCa by correlating immunohistochemical (IHC) and fluorescent *in situ* hybridization (FISH) with well-established prognostic values in PCa and with patients’ biochemical and pathological data and biochemical recurrence-free survival (BRFS). To the best of our knowledge, this is the first study to perform TOP2A protein and gene digital assessment and fractal analysis in association with BRFS and other clinical data in a large series of PCa.

## Patients and methods

### Tumor samples and biochemical and pathological data

Formalin-fixed paraffin-embedded tissue specimens from patients with PCa who underwent radical prostatectomy and were diagnosed at A.C. Camargo Cancer Hospital (Sao Paulo, Brazil) between 1991 and 2009 were retrieved for the study. As a result, a tissue microarray (TMA) containing 193 prostatic adenocarcinomas was constructed. Inclusion criteria were the availability of suitable paraffin blocks for IHC and follow-up information.

All PCa were graded based on the Gleason system by 2 independent pathologists at A.C. Camargo Cancer Hospital in a blind and consecutive manner to ensure adequate diagnosis and grade. The TNM staging system was used to describe the extent of PCa in patients (based on the AJCC Cancer Staging Manual, Seventh Edition, 2010, Springer New York, Inc.). TNM stages IIA and IIB were considered TNM stage II. All samples in this study were collected prior to hormone treatment or radiotherapy. The following biochemical and pathological parameters were recorded: preoperative PSA, Gleason score, TNM stage, lymph node status, angiolymphatic invasion, extraprostatic extension, margin status and seminal vesicle (SV) invasion. The study was approved by the ethics committee of our institution (Research Ethics Committee of A. C. Camargo Cancer Hospital) under process number 1473/10.

### Tissue microarray construction

Representative areas of prostatic adenocarcinoma were marked on hematoxylin- and-eosin-stained sections and cylinders 1 mm in diameter were punched from selected areas of the donor paraffin blocks (Beecher Instruments, Silver Spring, MD, USA). Each tumor was sampled twice in the TMA block that was cut 3 μm thick for the IHC and FISH studies.

### Immunohistochemistry (IHC)

IHC staining was performed using automated standard procedures (Flex Plus detection system, DAKO, Carpenteria, CA, USA) and mouse monoclonal antibody against TOP2A (SWT3D1 clone; DAKO, Carpenteria, CA, USA) at dilution 1:800. Specific standardized protocol supplied by the manufacturer was followed. Testis was used as positive control, and omission of primary antibody was applied as negative control.

### Immunohistochemical analysis

All slides were digitalized using Aperio System (Vista, CA, USA), and the images provided by the software were exhibited on an LCD monitor under contrast, focus, saturation, and white balance standardization. Automated image quantification was performed using the nuclei quantification algorithm, which associates staining intensity with percentage of stained cells and generates a final score ranging from 0 (negative) to 1 (weak positive), 2 (moderate positive), or 3 (strong positive). Then, numerical scores were exported to a Microsoft Excel (Seattle, WA, USA) file for further statistical analysis.

### Fluorescent *In situ* Hybridization (FISH)

FISH was performed using TOP2A (17q21)/CEP17 probe kit (Kreateck Biotechnology USA Inc, San Diego, CA, USA) and DAPI counterstaining. Fluorescent slides were assessed in a fluorescent microscope concerning chromosome 17 polyssomy and *TOP2A* gene amplification or deletion.

### Fractal analysis

20 PCa samples were randomly selected from our set to perform the fractal analysis. The histochemically stained cells were selected for capturing images and target stained cells were microphotographed in an isolated condition, with minimal contact or overlap of neighboring cells.

Cell images were captured with a Nikon D90 SLR digital camera 12.9 mega pixels DX-format and a file format of JPEG with an image size of 1,424-4,288 connected to the photoport attachment of a Nikon diaphot 300 inverted phase contrast microscope. Cells were observed with a 10×/20m wide field adjustable eyepiece at 400× magnification. Original images were stored in a TIFF format from JPEG in order to perform a background color correction using Micrografix picture publisher 8 windows graphic software. The images were then imported into Adobe Photoshop version CS5 for Macintosh. From each image, the three most suitable histochemically stained cells for fractal analysis were chosen. These three chosen cells were then cropped out of their original images with a 65 × 65 pixel frame that was centered on the chosen stained cell. Utilizing the quick selection tool in Photoshop (with settings of 100% hardness, 8% spacing, auto-enhance turned off, and a selection size of three pixels) secondary histochemically stained cells present in the targeted 65 × 65 pixel image were selected and cropped out, resulting in one stained cell per new created image without artifacts (i.e., fragments of other cells). Captured images were stored in BMP format, with a bit depth of 24, and were then binarized by the Benoit 1.3 fractal analysis software (TruSoft International Inc., St. Petersburg, FL) to calculate the fractal dimension of the cells.

For the fractal dimension calculation, box dimension was taken as an appropriate approximation of fractal dimension. In the present study due to the original JPEG dimensions and size of the cells, the box sizes were set at 65, and the fractal dimension was computed by using the Benoit software (TruSoft International Inc., St. Petersburg, FL).

### Statistical analysis

Mann–Whitney test was used to calculate the correlation between numerical variables. *X*^*2*^ test was used to evaluate differences in frequency of categorical-variable groups. Spearman’s rank correlation was used to analyze the correlation between continuous variables. Kaplan-Meier and log-rank tests were used to evaluate survival rates before biochemical recurrence concerning expression of TOP2A with BRFS (based on the increase in PSA, defined as PSA level > 0.2 ng/ml). The level of statistical significance was set to 0.05 for these tests. Statistical analyses were performed using SPSS, version 11.0 (USA).

## Results

### Patients and clinical data

102 patients (52.84%) presented Gleason score of 6, and the remaining 91 cases (47.16%) presented Gleason score between 8–10. 95 patients (49.22%) presented TNM stage II; 86 (44.55%) presented stage III; and 8 patients (6.23%) presented TNM stage IV. The median preoperative PSA level was 8.6 ng/ml. Other clinicopathological features are summarized in Table 1.

**Table 1:** Characterization of the cohort of 193 prostate cancer samples

**Characteristics**	**n(%)**
**Age (years)**	
< 50	11 (5.69)
50 - 70	160 (82.9)
>70	21 (11.4)
**Preoperative PSA (ng/ml)**	
< 10	114 (60.6)
10 ≥ PSA < 20	50 (26.5)
≥ 20	24 (12.9)
**TNM stage (pathologic)**	
II	95 (49.2)
III	86 (44.6)
IV	8 (6.23)
**Gleason score**	
6 (3 + 3)	102 (52.8)
7 (3 + 4)	46 (23.8)
7 (4 + 3)	19 (9.8)
8 - 10	26 (13.5)
**Angiolymphatic invasion**	
Presence	44 (23.2)
Absence	146 (76.9)
**Extraprostatic extension**	
Presence	41 (21.7)
Absence	148 (78.3)
**Positive margin**	
Presence	68 (21.6)
Absence	122 (78.4)
**Seminal vesicle invasion**	
Presence	22 (11.6)
Absence	168 (88.4)
**Positive lymph node**	
Presence	10 (5.44)
Absence	174 (94.6)
**Biochemical recurrence**	
Presence	115 (60.5)
Absence	75 (39.5)

PCa patients who had higher Gleason scores (*p* < 0.001), higher TNM stages (*p* < 0.001), positive margin (*p* = 0.002), SV (*p* < 0.001), angiolymphatic invasion (*p* = 0.019), and positive lymph nodes (*p* = 0.003) presented shorter BRFS. The median of BRFS in our set of PCa patients was 30.49 months, ranging from 0.3 to 108.88 months.

### IHC and FISH signals

**Figure 1:**
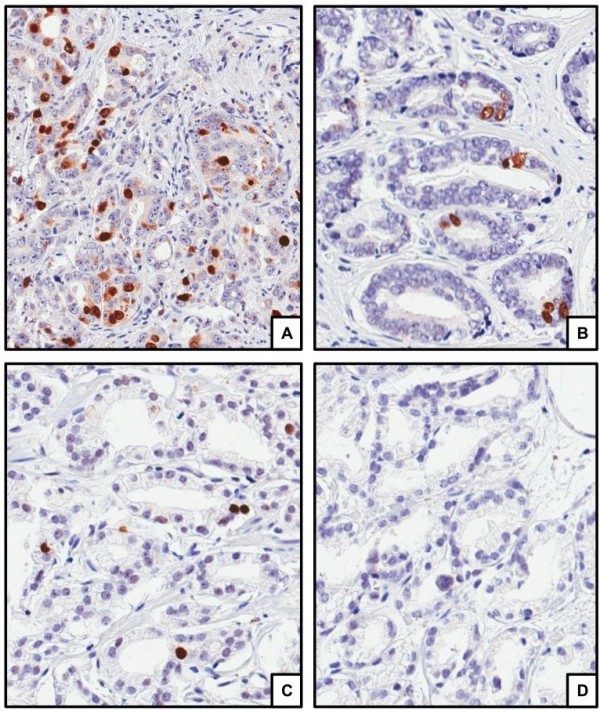
Immunostaining for TOP2A in prostate cancer observed at 200 magnification showing 3+ staining (A); 2+ staining (B); 1+ staining (C); and negative staining (D).

**Figure 2:**
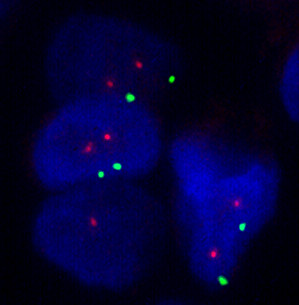
**Examples of fluorescent in situ hybridization for *TOP2A *gene (orange) and chromosome 17 (green) showing no copy number alteration.**

IHC sharp and specific nuclear staining for TOP2A was observed in tumor cells as shown in Figure 1. Orange signals for *TOP2A* gene and green signals for centromeric region of chromosome 17 were observed; however, no numerical alterations in *TOP2A* gene and chromosome 17 were noticed in any tumor sample (Figure 2).

### TOP2A and biochemical and pathological data

**Figure 3:**
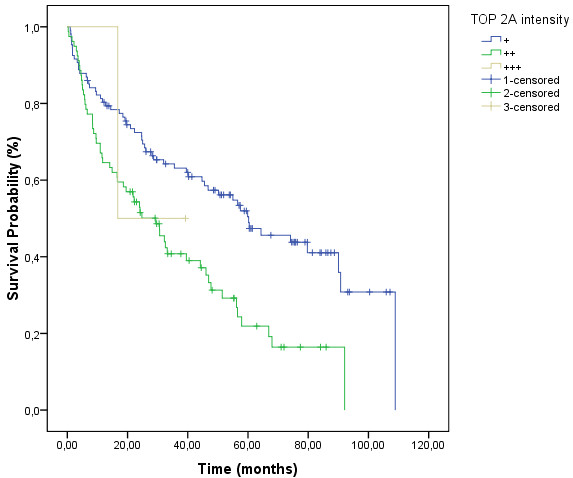
Biochemical recurrence-free survival curves showing lower survival rates of those cases which expressed high levels of TOP2A protein.

Patients who expressed higher levels of TOP2A presented higher Gleason scores and higher levels of preoperative PSA (*p* = 0.018 and *p* = 0.011, respectively). Also, patients with higher expression of TOP2A presented shorter BRFS (*p* = 0.001; Figure 3), as shown in Table 2. On the other hand, there was no difference between tumors with Gleason score 7(3 + 4) and tumors with Gleason score 7(4 + 3) concerning TOP2A IHC expression.

**Table 2:** **Association between TOP2A expression and biochemical recurrence**-**free survival**

**TOP2A intensity**	**Median(months)**	p*
1+ (weak)	60.230	0.001
2+ (moderate)	29.375	
3+ (strong)	16.743	

* Statistically significant *p* values inferior to 0.05.

TOP2A remained as an independent prognostic factor for BRFS in the multivariate analysis, with a relative risk of 1.98 (*p* = 0.001; 95% CI, 1.338 – 2.93). Thus, cases that expressed higher TOP2A presented shorter BRFS compared with TOP2A-negative or TOP2A-lower cases. The presence of SV invasion (hazard ratio = 3.038; *p* < 0.001; 95% CI, 1.755 – 5.256) also remained as an independent prognostic factor for BRFS (Table 3).

**Table 3:** **Multivariate analysis for biochemical recurrence**-**free survival**

**Variables**	**Hazard ratio**	**95%CI**	p*
TOP2A (higher intensities)	1.98	1.338 – 2.93	0.001
Seminal vesicle invasion (presence)	3.038	1.755 – 5.256	<0.001

* Statistically significant *p* values inferior to 0.05.

### Fractal analysis

**Figure 4:**
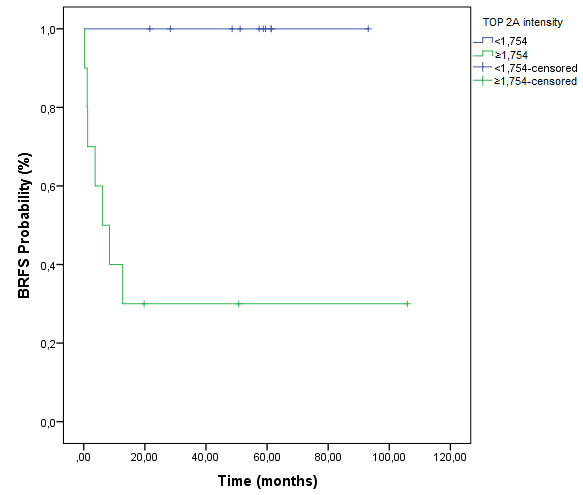
**Biochemical recurrence**-**free survival curves concerning fractal analysis of a subset of 20 randomly selected cases showing lower survival rates of those who expressed higher levels of TOP2A protein.**

Median of TOP2A intensity evaluated in a subset of 20 randomly selected cases was 1.754. Patients who expressed higher levels of TOP2A (TOP2A intensity ≥ 1.754) presented angiolymphatic invasion and higher Gleason scores (*p* = 0.033 and *p* = 0.025, respectively). Also, patients with higher expression of TOP2A (TOP2A intensity ≥ 1.754) presented shorter BRFS (*p* = 0.001; Figure 4).

## Discussion

Analysis of the cell kinetics of cancer cells *in situ* (for example, by Ki-67 antigen expression or mitotic counts) has been increasingly used to evaluate prognosis and/or biological behavior of various human malignancies [12]. TOP2A is a nuclear enzyme that controls DNA topological structure and cell cycle progression [13]. It mainly supports DNA decoiling, chromosome segregation during anaphase of the cell cycle, and DNA replication, by creating a DNA-linked protein gate through which another intact DNA duplex passes [14]. This enzyme is a marker of cell proliferation in normal and neoplastic tissues [15]. In malignant cells, overexpression of TOP2A protein might reflect not only the proliferative advantage of these cells, but also qualitative alterations caused by malignant transformation and dedifferentiation [12]. The IHC method for *in situ* determination of TOP2A has been extensively validated and shown to reflect closely the exact enzyme activity in formalin-fixed paraffin-embedded human tissues, leading to the prognostic and predictive importance of this test in other neoplasms [16].

This is the first study that brings together a gene and protein assessment of TOP2A in the largest series of prostate adenocarcinoma. Also, fractal image analysis was performed in order to confirm digital assessment in a subset of randomly selected cases. Furthermore, for the first time we bring an association between all these data and BRFS curve.

Concerning IHC pattern of expression for TOP2A, positive nuclear staining was found. Also, additional diffuse weak cytoplasmic staining was seen in some cases, as Faggad *et al*. [17] and Gotlieb *et al*. [18] also reported in their respective studies. Corroborating most works on literature, we found that the expression of TOP2A was an indicative of poor prognosis. Patients who expressed higher levels of its protein had higher Gleason scores, higher levels of preoperative PSA, and shorter BRFS. Indeed, high proliferation rates in cancers are typically associated with worse clinical outcome [4]. According to Faggad *et al*. [17], one explanation for the shorter survival rate associated with elevated TOP2A levels could be an enhancement of tumor cell proliferation, which results in increased tumor aggressiveness. In the multivariate analysis, TOP2A positivity remained an independent prognostic factor for BRFS, along with the presence of SV invasion.

Increased TOP2A is a common, though not specific, occurrence in malignant cells [19]. According to O’Connor *et al*. [19], there are several mechanisms to explain this upregulation in these cells. First, both pRB and p53 are negative regulators of topoisomerase IIα, and both are well known to be inactivated or deleted in malignant cells [14]. Inactivated or deleted pRB or p53 would be expected to increase the expression of this enzyme [19]. Another possible mechanism for the overexpression of TOP2A is the amplification of a coding gene such as that for HER2/*neu*[19]. Both HER2/*neu* and *TOP2A* reside on the long arm of chromosome 17 and amplification of one gene locus could simultaneously overexpress both of these genes [19]. In the present study, we did not find any alterations on *TOP2A* status in FISH. Schindlbeck *et al*. [14] found that *TOP2A* amplification was not significant for outcome in women with primary breast cancer, protein expression only (IHC) was related to outcome in those patients. According to this work, protein expression might be more relevant than *TOP2A* amplification or deletion in predicting the outcome of breast cancer patients that received anthracycline-based chemotherapy [14]. In another study of our group [20], *TOP2A* amplification did not correlate with FISH results in soft tissue sarcomas. According to Werneck *et al*. [20], increased TOP2A expression does not appear to result solely from gene amplification. The explanation for this finding is still unclear; it might be due to posttranscriptional regulation [20]. This corroborates studies on other solid tumors, like breast cancer [21,22] and gastric carcinoma [23]. Werneck *et al*. [20] suggested that gene amplification and protein expression should be evaluated separately when the prognostic or predictive value of TOP2A is examined in any neoplasia.

High levels of TOP2A expression are generally associated with high levels of cellular proliferation and poor histologic differentiation of tumors [24]. The relationship between overexpression of this enzyme and poor prognosis has been reported in different neoplasias, like breast cancer [19], urothelial bladder carcinoma [12], larynx cancer [15], bladder cancer [24], and ovarian cancer [17]. On the other hand, although most studies on literature correlates high levels of TOP2A expression with poorer survival rates and more aggressive tumors, there are studies showing the opposite. Bredel *et al*. [25] concluded that high expression of TOP2A and Ki-67 appeared to be associated with prolonged survival in glioblastoma patients. In a recent work, Schindlbeck *et al*. [14] showed that TOP2A IHC positivity predicted lower risk of metastases and death in breast cancer patients. Yan *et al*. [26] demonstrated that high TOP2A expression was correlated with better disease-free survival for postoperative non-small cell lung cancer (NSCLC) patients who received adjuvant chemotherapy. One reason for these discrepant results is that these patients received adjuvant chemotherapy and high grade tumors tend to present better response to this type of therapy. Yan *et al*. [26] postulated that adjuvant chemotherapy might overcome the adverse biology of cancers that expressed high levels of TOP2A protein. According to this latter work, NSCLC patients with high expression of the enzyme might be able to obtain more benefits from adjuvant chemotherapy than those with low expression, which emphasizes the predictive importance of TOP2A for such patients [26].

Proliferation measurements in PCa have generally been done by studying the Ki-67 molecule, which is present in actively cycling cells [5]. IHC for Ki-67 in PCa has been shown to have prognostic importance (tumors with high Ki-67 expression tend to have a poorer prognosis and high tumor Ki-67 value also appear to predict tumor recurrence after radical prostatectomy) [5]. Since TOP2A has been found to correlate well with Ki-67 in a number of human diseases, Willman *et al*. [5] suggested that similar prognostic information might be obtained by TOP2A IHC, with an advantage that the enzyme is the target of drugs being used for treating PCa patients. However, there are few works in the literature correlating TOP2A and clinicopathological parameters of PCa. Sullivan *et al*. [7] showed that the expression of this enzyme increased with both stage and grade, and advancing stage was the stronger predictor of TOP2A expression. Willman *et al*. [5] and Hasby *et al*. [8], in their respective works, demonstrated that the prostatic carcinomas with the highest expression of the enzyme were more poorly differentiated and had the highest Gleason scores. Hughes *et al*. [6] showed that TOP2A expression increased with increasing Gleason score and with hormone insensitivity. Murphy *et al*. [9] showed that increased *TOP2A* copy number was associated with adverse clinical features, including high Gleason score, high stage, androgen resistant, *HER2* amplification, and decreased survival under multivariate analysis. In a recent study, Karnes *et al*. [10] demonstrated that the time for PCa patients to develop systemic progression (SP) was significantly associated with TOP2A protein expression: higher 5-year SP rates were observed in patients with higher protein levels of the enzyme. Ida *et al*. [11] showed that TOP2A protein expression was predictive of SP and death in PCa patients with Gleason score ≥7 treated surgically, especially in PCa without ERG overexpression. Malhotra *et al*. [4] demonstrated that a tri-marker proliferation index (which included Ki-67, TOP2A, and E2F1) provided improved prognostic performance in PCa; it predicted biochemical recurrence after radical prostatectomy.

Alenda *et al*. [27] showed that PCa patients with Gleason score 7(4 + 3) have higher chances of presenting biochemical recurrence compared to patients with Gleason score 7(3 + 4). In addition, they also showed that the primary Gleason pattern 4 remained as an independent prognostic factor for the occurrence of biochemical recurrence in PCa patients [27]. Although the clinical difference between patients from both groups of Gleason 7 [(3 + 4) and (4 + 3)] is largely known, we found no difference in TOP2A expression between them. We suppose that TOP2A may not be related (at least not by itself) to early stages of carcinogenesis and morphological undifferentiating, which may result in further aggressive tumor biological behavior. However, when analyzing all Gleason scores there was a significant difference in TOP2A expression, showing that this protein may come up in more discrepant lesions. Furthermore, the absolute relation between the presence of TOP2A in tumors of patients with biochemical recurrence is an evidence that this protein is related to late stages of tumor development.

Besides its important role as a proliferation marker, as shown above, TOP2A is also the molecular target of several chemotherapy agents, including anthracyclines such as doxorubicin and etoposide [19]. These cytotoxic agents bind the DNA topoisomerase II complex and inhibit the relegation of DNA [19]. This converts TOP2A into a physiologic toxin and introduces high levels of permanent double-stranded breaks, which are detected by proteins ensuring genomic integrity [28]. As a result of the activation of this machinery, the cells with abundant DNA breaks are eliminated by apoptosis [28]. The sensitivity or resistance of a malignant cell to these anti tumor drugs, also called topo II poisons, is proportional to the level of TOP2A expression [11,14]. In PCa, there are several phase II trials using etoposide or docetaxel in combination with other chemotherapy agents to treat androgen-sensitive metastatic prostate carcinoma and hormone-refractory PCa, with promising results [29-31].

The biological behavior of PCa still challenges researchers and urologists. While some patients present indolent disease with no need for treatment, others present aggressive disease with inevitable progression. Therefore a good prognostic and predictive marker of adjuvant and target therapies would be of great utility. In this sense, TOP2A is emerging as an important molecular target for many anticancer drugs, and several experimental works have clearly showed that cellular sensitivity to this enzyme is dependent on its high levels [30]. In conclusion, we found that higher expression of TOP2A protein in PCa patients is a strong indicative of poor prognosis. Also, since TOP2A is a target for many anti-neoplastic drugs, the IHC evaluation of this marker in routine practice can be a powerful tool for selecting appropriately aggressive therapies (use of adjuvant chemotherapy), specific target therapies, and the most suitable surgery approach in order to improve outcome of patients with prostate cancer. Also, we show for the first time that *TOP2A* gene copy number alterations are not observed in this type of tumor. So, higher protein expression of TOP2A is not related to gene amplification in PCa. Furthermore, TOP2A protein assessment has prognostic importance and, due to its relation with poor outcome, TOP2A IHC evaluation in the biopsy can represent an important tool for selecting the most suitable surgical and clinical approach for patients with PCa.

## Competing interests

The authors have declared no conflicts of interest.

## Authors’ contributions

MFR wrote the manuscript and participated in the data analysis and interpretation. SV helped to draft the manuscript. LTDC performed the statistical analysis. FC, PAP, AB and XB performed the fractal analysis. FPF and GCG participated in the provision of study material or patients, and in the collection and assembly of data. FAS provided the financial support, provision of study material or patients and administrative support. IN and SP carried out the immunoassays. RMR participated in the conception and design, and in the data analysis and interpretation, provided the financial support and the final approval of manuscript. All authors read and approved the final manuscript.
